# Multimodal deep learning for prediction of postoperative recurrence in clear cell renal cell carcinoma: a clinical-radiologic-pathologic approach

**DOI:** 10.3389/fonc.2026.1831252

**Published:** 2026-08-06

**Authors:** Youchang Yang, Jiaojiao Wu, Feng Shi, Qingguo Ren, Peng Gao, Xiangshui Meng

**Affiliations:** 1 Department of Radiology, Qilu Hospital (Qingdao), Cheeloo College of Medicine, Shandong University, Shandong, China; 2 Department of Research and Development, United Imaging Intelligence, Shanghai, China; 3 Department of Radiology, Qilu Hospital, Shandong University, Jinan, China

**Keywords:** clear cell renal cell carcinoma, multi-classifier system, prediction, radiomics, recurrence

## Abstract

**Purpose:**

This study intends to develop and validate a multiclass system that efficiently integrates complementary information from patients’ multimodal data (clinical, imaging, and pathological) to identify individuals at a high risk of 5-year postoperative recurrence among patients with clear cell renal cell carcinoma (ccRCC).

**Methods:**

This retrospective multicenter study enrolled 270 clear cell renal cell carcinoma (ccRCC) patients, allocating 215 (79.6%) for model development/validation and 55 (20.4%) to an independent test cohort. Patients were categorized into recurrence and non-recurrence groups based on whether tumor recurrence occurred within 5 years after surgery. The final included case in this cohort underwent surgery in July 2018. Single-modality models were created using radiomics, deep learning (ResNet34), and deep features-based radiomics. Subsequently, multi-modality models were constructed by combining the predicted probabilities from the single-modality models and transferring them to another classifier. All models was assessed using the area under the receiver operating characteristic curve (AUROC).

**Results:**

A total of 25 classification models were constructed. Notably, single-modality fusion models generally outperform their radiomics and deep learning-based radiomics (DL) counterparts. Among five single-modality fusion models, the Fused_Non-enhanced model demonstrates best predictive performance, achieving an AUROC value of 0.837 (95% confidence interval [CI]: 0.729-0.946). Similarly, multi-modality radiomics or DL models exhibit superior performance compared to single-modality counterparts. The multi-modality radiomics-DL model demonstrates the highest prediction performance, achieving an AUROC value of 0.967 (95% CI: 0.929-1.0) in the independent testing dataset.

**Conclusion:**

The multi-modality radiomics-DL model demonstrates high accuracy in predicting the 5-year postoperative recurrence risk of clear cell renal cell carcinoma (ccRCC).

## 1 Introduction

Renal cell carcinoma (RCC) is the most prevalent type of kidney cancer, and ccRCC serves as the predominant subtype (1–3). Radical nephrectomy remains the primary curative treatment for localized ccRCC; however, postoperative tumor recurrence constitutes a major clinical hurdle that severely impairs long-term patient prognosis. Epidemiological data indicate that approximately 20%–40% of ccRCC patients experience tumor recurrence within five years following surgical resection (4, 5). For patients at moderate-to-high or high risk, adjuvant immunotherapy is recommended (6, 7). Consequently, precise prediction of postoperative recurrence risk is paramount, providing a critical basis for individualized adjuvant treatment decision-making and rational clinical management.

Artificial intelligence (AI) in medical imaging has emerged as an indispensable tool, significantly enhancing diagnostic accuracy and decision-making processes. Radiomics is a high-throughput quantitative analysis approach that extracts hundreds to thousands of handcrafted or deep learning-derived features (e.g., intensity, texture, shape, and spatial relationship) from standard clinical medical images (such as CT, MRI, and ultrasound) through computational algorithms. By transforming visual imaging information into objective, reproducible quantifiable features, radiomics enables a comprehensive characterization of intratumor and intertumoral heterogeneity that is not visually perceptible to radiologists (7, 8). This technique has achieved promising outcomes in diagnosis (9, 10), tumor grading (11, 12), and prognosis (13, 14). Notably, each data modality offers unique information: clinical data (e.g., demographics, smoking/drinking history, laboratory indices) captures systemic patient status and long-term exposure factors; imaging data enables non-invasive assessment of macroscopic features (e.g., size, necrosis, vascular invasion, spatial distribution); and pathological data (H&E slides) reveals microscopic biological essence (e.g., cell differentiation, nuclear grade, tumor microenvironment). However, single-omics cancer data offers only limited insights, necessitating the integration of multi-omics cancer data to fully capture the underlying biological information (15–20). The growing availability of multi-omics cancer data has spurred efforts to integrate these diverse data types, leading to the development of several survival and risk prediction models (21–24). Multi-omics machine learning (ML) approaches have demonstrated superior performance compared to traditional single-omics ones. Each approach has its unique strengths and limitations, and the selection of effective methods should be carefully tailored to the specific research objectives and available data types (25). To our knowledge, there remains a gap in studies that incorporate multi-omics information into the prediction of postoperative recurrence risk in ccRCC.

Therefore, this study aimed to explore the performances of different AI techniques in predicting the postoperative recurrence risk of ccRCC patients by incorporating multi-omics complementary information including demographic characteristics, laboratory tests indices, ccRCC radiological characteristics, ultrasound (US) images, three-phase CT scans, and histopathological (HP) sections.

## 2 Materials and methods

### 2.1 Participant recruitment

This retrospective multicenter study consecutively enrolled non-metastatic ccRCC patients at radical or partial nephrectomy between April 2013 and July 2018 at three different hospitals: A, B, and C. Patients had complete multimodal data, including three-phase CT, ultrasonography, histopathological WSIs, and clinical records. Missing data were handled using a complete-case strategy at the patient level (patients with incomplete multimodal data were excluded), and no clinical variable imputation was required. The detailed criteria for inclusion and exclusion are mentioned in **Supplementary Methods**.

**Figure 1:**
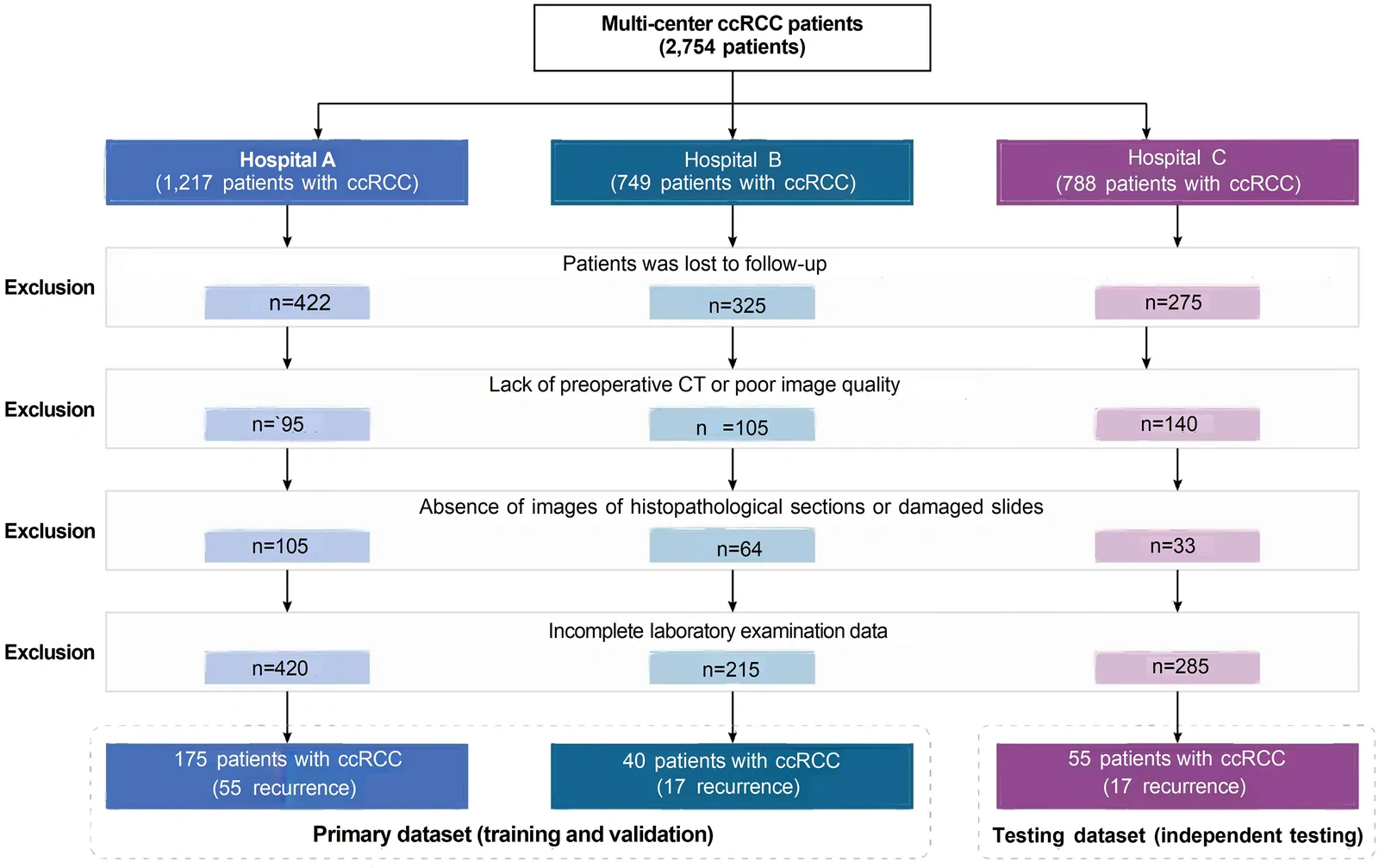
Preprocessing and filtering of multicenter ccRCC datasets. Diagram describing the inclusion and exclusion in this study.

Patients were divided into recurrence and non-recurrence groups according to their status five years after surgery. The recurrence group consisted of local recurrence and distant recurrence cases, all of which were confirmed by pathological biopsy. The inclusion and exclusion criteria are outlined in **Figure 1**. Excluded were those who died without recurrence within this period. Finally, 270 eligible patients remained (89 recurrence, 181 non-recurrence). Primary datasets included 215 patients from hospitals A and B (training/validation), while independent testing 55 patients from hospital C. To justify this sample size, a statistical power analysis was performed based on the predictive performance (AUC) of the final multi-modality fusion model. Under a two-sided Type I error rate (α) of 0.05 and the actual ratio of recurrence to non-recurrence patients of 1:2.03 (89/181), our total cohort of 270 patients provides a statistical power of 93.1% to detect a conservative target AUC of 0.80 against a null hypothesis AUC of 0.50 (calculated using the Obuchowski-McClish method for ROC curves; see **Supplementary Figure 1** for the power curve). This confirms that our multi-tiered study is robustly powered for both model development and evaluation.

### 2.2 Imaging acquisition

#### 2.2.1 CT and US scanning

All participants underwent standard three-phase CT and two-dimensional ultrasound. CT was done with a multi-detector spiral CT scanner at each center, covering the whole kidney. The ultrasound examinations were performed with a commercial ultrasound system. The detailed acquisition protocol and scanning parameters of the different centers are given in **Supplementary Table 1**. Image quality was stringently assessed by the radiologist and sonographer, and any disputes were resolved by senior expert consensus.

#### 2.2.2 HP staining and scanning

Surgical tissue specimens were fixed in formalin, embedded in paraffin, and sectioned for H&E staining. Representative tumor sections were digitized as WSIs at 20× magnification (0.5 μm/pixel) using a slide scanner, which were independently reviewed by two genitourinary pathologists. Slides with extensive necrosis, hemorrhage, fixation artifacts, or insufficient tumor cellularity were excluded. One representative slide per case was selected for digitization and subsequent annotation. The representative slide was chosen to correspond to the region with the highest tumor cellularity and the highest histologic grade (according to the [e.g., WHO/ISUP] grading system), in accordance with the predominant pathological features of the lesion. Within the selected slide, the area of highest cellularity and highest grade was manually delineated for annotation; the remaining portions of the slide were not annotated. Any disagreement between the two pathologists was resolved by consensus review to ensure consistency in both slide and region selection.

### 2.3 Image preprocessing and annotation

This module describes the standardized preprocessing carried out for the different modalities to ensure consistency in the data. That is, for CT, preprocessing included resampling, gray-level normalization, and discretization; US included denoising and contrast enhancement; and for HP, Macenko color normalization was used to standardize the staining variation, followed by noise reduction and patch extraction. Further details on the preprocessing algorithms and parameters used (e.g., stain vectors) can be found in the **Supplementary Methods**.

#### 2.3.1 Annotation of CT data and construction of ccRCC segmentation model

Two radiologists manually delineated 3D ROIs that included the entire tumor volume in the cortical enhancement phase CT images. These ROIs were propagated using rigid registration (quality checked via Dice coefficient > 0.80). To explore the feasibility of future clinical automation, we developed an automatic ccRCC segmentation pipeline using nnU-Net, which achieved high segmentation accuracy (detailed in **Supplementary Table 2**). However, to ensure maximum precision and consistency in prognostic performance during this phase of the study, all downstream feature extraction and model development were conducted using only the manual ROIs.

#### 2.3.2 Annotation of US and HP data

Firstly, for US data, two sonographers selected the tumor slice with the greatest cross-section, then manually outlining the 2D ROIs excluding normal parenchyma. Second, in HP data, two pathologists manually annotated the tumor core area (excluding necrosis) in WSIs using ImageScope software. From the annotated areas, non-overlapping patches 256×256 pixels were sampled for feature extraction. Third, inter-observer variability was maintained across all modalities.

### 2.4 Model development

#### 2.4.1 Experimental design and data grouping

**Figure 2:**
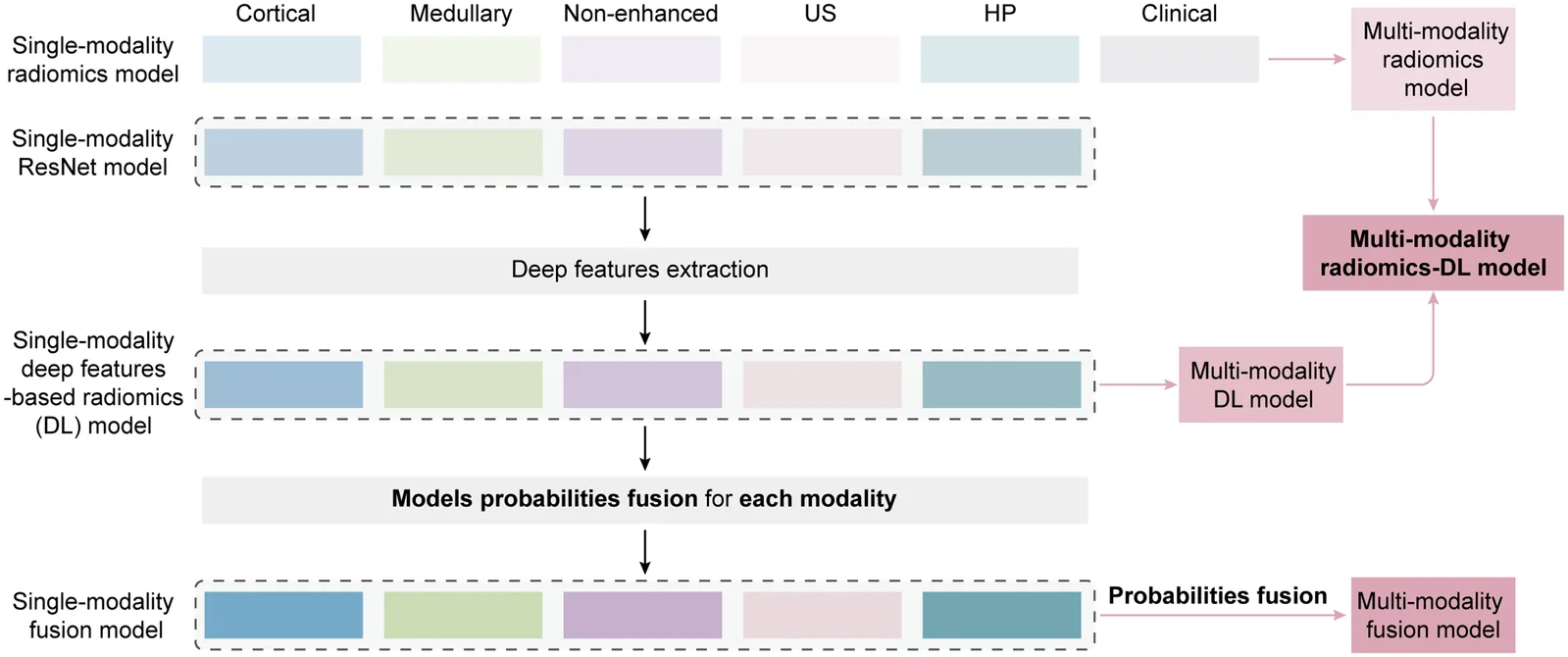
Experimental design and models construction in this study.

Accordingly, we hierarchically structured a modeling system from single-modality models to multi-modality models to predict the postoperative recurrence of ccRCC (**Figure 2**). Patients from three medical centers were divided into two cohorts: a primary dataset from Hospitals A and B combined (n=215) for model development, and an independent testing cohort from Hospital C (n=55) for external evaluation. During model development, different validation schemes were tailored to the algorithm requirements. Specifically, the radiomics models underwent feature selection and hyperparameter tuning using 5-fold cross-validation on the primary dataset. For the ResNet-based deep learning models, the primary dataset was split into a fixed training set (80%) and a validation set (20%) to facilitate the early stopping process. Finally, fusion models were constructed using a stacking ensemble framework, where predicted probabilities from the base models served as meta-features for a Logistic Regression meta-classifier. To strictly prevent data leakage and optimistic bias, these meta-features for the primary dataset were generated using out-of-fold predictions during the 5-fold cross-validation of the base models. Therefore, we developed four model tiers: single-modality radiomics (handcrafted features + ML), single-modality ResNet (end-to-end DL), deep features-based radiomics (DL) (ResNet features + ML), and fusion models (stacking ensemble). Fusion models were built within a stacking ensemble framework whereby predicted probabilities from base models acted as meta-features for a meta-classifier (Logistic Regression). This was a process that led to the development of single-modality fusion models (Radiomics and DL for one modality) and multi-modality fusion models (across modalities). Of notable consideration, meta-classifiers were exclusively trained on the primary dataset to mitigate against data leakage. In all, 25 models were constructed.

#### 2.4.2 Model construction

1. Single-modality radiomics models: The radiomics pipeline was implemented using the uAI research portal (uRP, United Imaging Intelligence) (26) and PyRadiomics in compliance with IBSI standards (27). Whole-tumor 3D ROIs were used for feature extraction from three-phase CT (2264 features/phase), 2D ROIs for US (1899 features), and tumor core patches for HP (93 features) and 41 clinical characteristics. The primary dataset underwent 5-fold cross-validation for feature selection. High-stability features (ICC > 0.75) were screened using LASSO regression or F-tests. To avoid overfitting, the final feature number was constrained to six per model under the 10 events per variable rule. A variety of preprocessing steps (e.g., feature scaling and class-imbalance handling) and machine learning classifiers (logistic regression, random forest, and boosting trees) were compared for establishing single-modality models.
2. Single-modality ResNet models and deep features-based radiomics models: A ResNet34 architecture (28) was used that had been initialized with pre-trained weights. Images were resized to 256×256, normalized, and concatenated with ROI masks as dual-channel inputs. Models were trained using cross-entropy loss optimized by Adam (learning rate = 1×10^−4^ with a weight decay of 1×10^−5^ for L2 regularization). To limit overfitting and enhance reproducibility, we applied online data augmentation (including random horizontal or vertical flips and rotations), integrated a dropout rate of 0.5 in the dense layers, and implemented an early stopping mechanism with a patience of 10 epochs based on the validation loss. For deep features-based radiomics models, deep features were extracted from the penultimate fully connected layer from the trained ResNet. Then, those features went through the same selection and classification pipeline as specific radiomics models, LASSO selection followed by machine learning classification, to yield predictions.
3. Single-modality fusion models: A stacked ensemble approach was applied here. Probabilities from the optimized single-modality radiomics models and their corresponding deep features-based radiomics models were used as meta-features. The meta-features, deriving from the primary dataset, were fed into the meta-classifier to output the final recurrence probability. Notably, this is where the stacked ensemble prevents data leakage, as all data used to construct the stacked ensemble are kept separate from the independent test set.
4. Multi-modality fusion models: Building on the same stacked ensemble framework, these higher-level models were fitted to combine information across modalities. The Multi-modality Radiomics model combined probabilities from the six single-modality radiomics models. The Multi-modality DL model combined probabilities from all five single-modality DL models. The Multi-modality Fusion model combined predictions from all five single-modality fusion models that were each modality Radiomics + DL. Finally, the Multi-modality Radiomics-DL model combined the outputs of the Multi-modality Radiomics and Multi-modality DL models hierarchically. In this stacking framework, continuous prediction probabilities (P∈[0,1]) from base models were utilized as meta-features. Unlike simple score-level averaging, this hierarchical stacking uses a Logistic Regression meta-classifier to adaptively learn optimal weights for each modality’s output, capturing non-linear interactions while minimizing noise from less reliable models.

So far, all meta-classifiers have been trained on the primary dataset. The testing dataset is solely independent and has been reserved for Hospital C to assure no data leakage occurs. Please consult the **Supplementary Methods** for the detailed model naming conventions.

### 2.5 Model evaluation

The optimized models were validated on the independent testing dataset. The classification performance was estimated via ROC curves and AUROC, with the 95% confidence intervals derived from 1000 bootstrap iterations. Other analyses included PR curves and AUPR. Quantitative metrics like accuracy, sensitivity, specificity, precision, and F1-score were also computed to check whether the prediction was consistent.

### 2.6 Statistical analysis

Continuous variables were assessed using ANOVA or Kruskal-Wallis H test, depending on normality, as assessed by the Shapiro-Wilk test. Categorical variables were assessed using Chi-square tests. Univariate logistic regression was performed to determine significant risk factors. For model performance comparison, AUROCs were determined across 1000 bootstrap intervals using R (fbroc package). Differences between multi-modality models and their respective single-modality or fusion baselines were determined using Kruskal-Wallis H tests, followed by Dunnett’s multiple comparisons tests. Statistical significance was considered at a two-tailed p < 0.05. All were analyzed with SPSS (v26.0) and R (v4.2.2). Detailed software packages and plotting tools are listed in **Supplementary Methods**.

## 3 Results

### 3.1 Patient population characteristics

The study included 270 ccRCC patients (194 males and 76 females). As shown in **Table 1**, significant differences were observed in 14 clinical variables across the three centers, reflecting population heterogeneity (**Supplementary Table 1**). Univariate logistic regression identified five significant risk factors for recurrence: maximum tumor diameter, arteriovenous thrombosis, lymphadenopathy, necrosis, and drinking (defined as a binary history of regular alcohol consumption prior to surgery) (**Supplementary Table 2**). These findings align with previous literature (29), confirming that clinic-radiological characteristics are valuable indicators for distinguishing recurrence.

**Table 1:** Patient clinical characteristics.

Variables	Hospital A	Hospital B	Hospital C	*P* value
No. of patients	175	40	55	–
Patient demographics				
Age (years)	60.00 (52.00, 66.00)	61.50 (53.00, 68.00)	63.00 (55.50, 69.50)	0.106
Sex (male, %)	127 (72.57%)	32 (80.00%)	35 (63.64%)	0.203
Height (cm)	170.00 (161.00, 173.00)	170.00 (161.75, 174.00)	168.00 (160.00, 170.50)	0.176
Weight (kg)	70.00 (62.50, 79.50)	68.50 (62.75, 80.50)	70.00 (60.50, 76.00)	0.914
Hypertension (n, %)	77 (44.00%)	18 (45.00%)	28 (50.91%)	0.667
Diabetes (n, %)	33 (18.86%)	7 (17.50%)	7 (12.73%)	0.579
Smoking (n, %)	60 (34.29%)	14 (35.00%)	8 (14.55%)	**0.017**
Drinking (n, %)	67 (38.29%)	16 (40.00%)	1 (1.82%)	**< 0.001**
Pain (n, %)	32 (18.29%)	6 (15.00%)	11 (20.00%)	0.820
Urination habits (n, %)	27 (15.43%)	6 (15.00%)	2 (3.64%)	0.070
Hematuria (n, %)	30 (17.14%)	8 (20.00%)	3 (5.45%)	0.071
Biochemical indicators				
Immunoglobulin	25.80 (23.40, 29.10)	26.60 (23.62, 29.27)	26.65 (25.33, 29.55)	0.233
Blood glucose	5.13 (4.80, 5.92)	5.14 (4.84, 6.62)	5.88 (5.25, 7.19)	**0.001**
Uric acid	305.00 (268.50, 352.50)	332.00 (287.00, 391.00)	338.00 (248.30, 404.20)	0.291
Blood urea nitrogen	5.20 (4.42, 6.10)	5.30 (4.22, 5.93)	5.50 (4.70, 6.65)	0.205
Creatinine	75.00 (64.00, 87.50)	78.00 (68.50, 89.50)	83.30 (67.90, 101.30)	0.083
White blood cells	6.21 (5.22, 7.68)	6.11 (5.18, 7.41)	7.16 (5.85, 9.88)	**0.006**
Neutrophils	3.85 (3.15, 5.16)	4.08 (3.23, 5.95)	4.81 (3.90, 7.82)	**0.002**
Lymphocytes	1.51 (1.17, 1.93)	1.46 (1.03, 1.88)	1.41 (0.96, 1.92)	0.486
Basophils	0.03 (0.02, 0.04)	0.03 (0.02, 0.03)	0.01 (0.01, 0.02)	**< 0.001**
Eosinophils	0.09 (0.05, 0.16)	0.08 (0.04, 0.11)	0.07 (0.04, 0.16)	0.271
Monocytes	0.48 (0.38, 0.60)	0.47 (0.37, 0.61)	0.49 (0.40, 0.70)	0.725
Red blood cells	4.57 (4.03, 4.98)	4.54 (4.04, 4.73)	4.30 (3.91, 4.77)	0.093
Hemoglobin	138.00 (120.50, 150.00)	137.00 (123.25, 147.25)	131.00 (113.50, 141.50)	0.078
Packed cell volume	41.20 (36.55, 44.75)	40.10 (37.97, 43.10)	38.30 (35.55, 41.60)	**0.025**
Mean corpuscular volume	90.20 (86.45, 93.25)	90.05 (86.62, 93.45)	88.90 (86.60, 92.20)	0.315
Platelets	227.00 (184.00, 276.50)	226.00 (185.25, 262.50)	196.00 (158.50, 237.50)	**0.016**
Urinary white blood cells	2.80 (1.40, 10.20)	2.35 (1.20, 10.35)	6.00 (3.00, 22.50)	0.070
Urinary red blood cells	5.70 (2.10, 15.40)	4.70 (1.87, 15.20)	11.00 (0.50, 24.50)	0.665
Epithelial cells	1.60 (0.80, 5.75)	2.70 (1.23, 8.00)	1.00 (0.00, 3.00)	**0.004**
Urinary occult blood (n, %)	56 (32.00%)	14 (35.00%)	23 (41.82%)	0.408
Abnormal urinary albumin (n, %)	30 (17.14%)	9 (22.50%)	13 (23.64%)	0.484
ccRCC characteristics				
Recurrence (n, %)	55 (31.43%)	17 (42.50%)	17 (30.91%)	0.380
Location (n, %)				0.328
• Left	81 (46.29%)	17 (42.50%)	31 (56.36%)	–
• Right	94 (53.71%)	23 (57.50%)	24 (43.64%)	–
Maximum diameter (mm)	4.00 (2.40, 6.50)	4.80 (2.00, 8.50)	4.00 (3.20, 5.10)	0.674
Arteriovenous thrombosis (n, %)	17 (9.71%)	9 (22.50%)	5 (9.09%)	0.060
Hemorrhage (n, %)	10 (5.71%)	4 (10.00%)	14 (25.45%)	**< 0.001**
Necrosis (n, %)	24 (13.71%)	6 (15.00%)	42 (76.36%)	**< 0.001**
Lobulation (n, %)	9 (5.14%)	3 (7.50%)	12 (21.82%)	**0.001**
Lymphadenopathy (n, %)	25 (14.29%)	11 (27.50%)	1 (1.82%)	**0.001**
Capsule (n, %)	1 (0.57%)	1 (2.50%)	45 (81.82%)	**< 0.001**
Calcification (n, %)	10 (5.71%)	4 (10.00%)	8 (14.55%)	0.101

A series of clinical features are compared among three hospitals using Kruskal-Wallis *H* tests or chi-squared tests. A two-tailed *P* value < 0.05 is considered significant difference. Bold values denote statistically significant clinical characteristics.

### 3.2 Automatic segmentation model for ccRCC

An nnU-Net model was constructed for automatic segmentation, achieving a high Dice Similarity Coefficient (DSC) of 0.897 in the testing dataset, indicating strong alignment with ground truth (**Supplementary Figure 2**; **Supplementary Table 3**). While this demonstrates potential for clinical automation, manual ROIs were used exclusively in this study to ensure strict consistency across multi-center data and modalities.

### 3.3 Construction and validation of classification models

#### 3.3.1 Single-modality radiomics models

**Figure 3:**
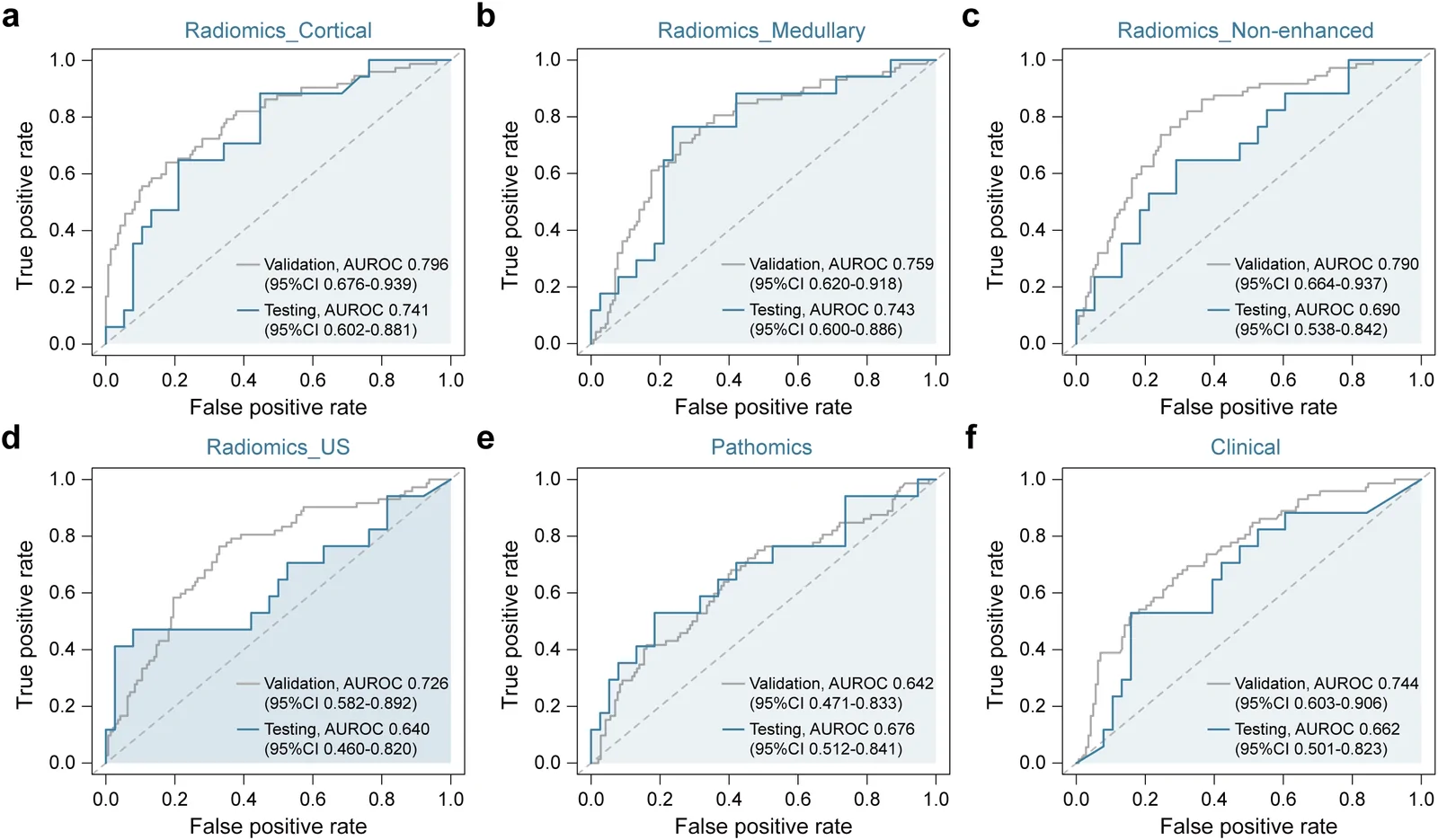
Classification performance of six single-modality radiomics models in validation and independent testing datasets. ROC curves for six radiomics models, including **(a)** Radiomics_Cortical, **(b)** Radiomics_Medullary, **(c)** Radiomics_Non-enhanced, **(d)** Radiomics_US, **(e)** Pathomics, and **(f)** Clinical models.

Radiomics features were extracted from three-phase CT, US, and HP images, alongside clinical data. After feature selection (**Supplementary Figure 3**; **Supplementary Table 4**), six single-modality models were constructed. In the independent test dataset, the Radiomics_Cortical model demonstrated the most favorable predictive performance (AUROC = 0.741), while the US-based model performed lowest (AUROC = 0.640) (**Figure 3**; **Table 2**).

**Table 2:** Classification performance of single-modality models in the independent testing dataset.

Model	AUROC (95% CI)	AUPR	ACC	F1	SEN	SPE	PRE
Radiomics models							
• Radiomics_Cortical	0.741 (0.602 – 0.881)	0.568	0.745	0.611	0.647	0.789	0.579
• Radiomics_Medullary	0.743 (0.600 – 0.886)	0.592	0.727	0.595	0.647	0.763	0.550
• Radiomics_Non-enhanced	0.690 (0.538 – 0.842)	0.557	0.673	0.526	0.588	0.711	0.476
• Radiomics_US	0.640 (0.460 – 0.820)	0.606	0.782	0.571	0.471	0.921	0.727
• Radiomics_HP	0.676 (0.512 – 0.841)	0.582	0.727	0.545	0.529	0.816	0.562
• Radiomics_Clinical	0.662 (0.501 – 0.823)	0.457	0.745	0.562	0.529	0.842	0.600
ResNet models							
• ResNet_Cortical	0.719 (0.575 – 0.862)	0.581	0.691	0.540	0.588	0.737	0.500
• ResNet_Medullary	0.715 (0.570 – 0.859)	0.500	0.691	0.452	0.412	0.816	0.500
• ResNet_Non-enhanced	0.728 (0.587 – 0.868)	0.507	0.709	0.579	0.647	0.737	0.524
• ResNet_US	0.647 (0.486 – 0.808)	0.440	0.582	0.511	0.706	0.526	0.400
• ResNet_HP	0.714 (0.563 – 0.864)	0.613	0.691	0.514	0.529	0.763	0.500
Deep features-based radiomics models (DL models)							
• DL_Cortical	0.741 (0.626 – 0.857)	0.506	0.691	0.667	1	0.553	0.500
• DL_Medullary	0.755 (0.626 – 0.885)	0.582	0.727	0.634	0.765	0.711	0.542
• DL_Non-enhanced	0.783 (0.657 – 0.910)	0.623	0.745	0.632	0.706	0.763	0.571
• DL_US	0.684 (0.512 – 0.856)	0.521	0.764	0.629	0.647	0.816	0.611
• DL_HP	0.801 (0.678 – 0.924)	0.708	0.782	0.667	0.706	0.816	0.643

#### 3.3.2 Single-modality ResNet models

Five ResNet34-based models were developed. The ResNet_Non-enhanced model achieved the optimal performance in the testing dataset (AUROC = 0.728), whereas the ResNet_US model showed inferior performance (AUROC = 0.647) (**Figure 3**; **Table 2**). Overall, the non-enhanced model demonstrated superior predictive capability compared to other single-modality ResNet models.

#### 3.3.3 Deep features-based radiomics models

**Figure 4:**
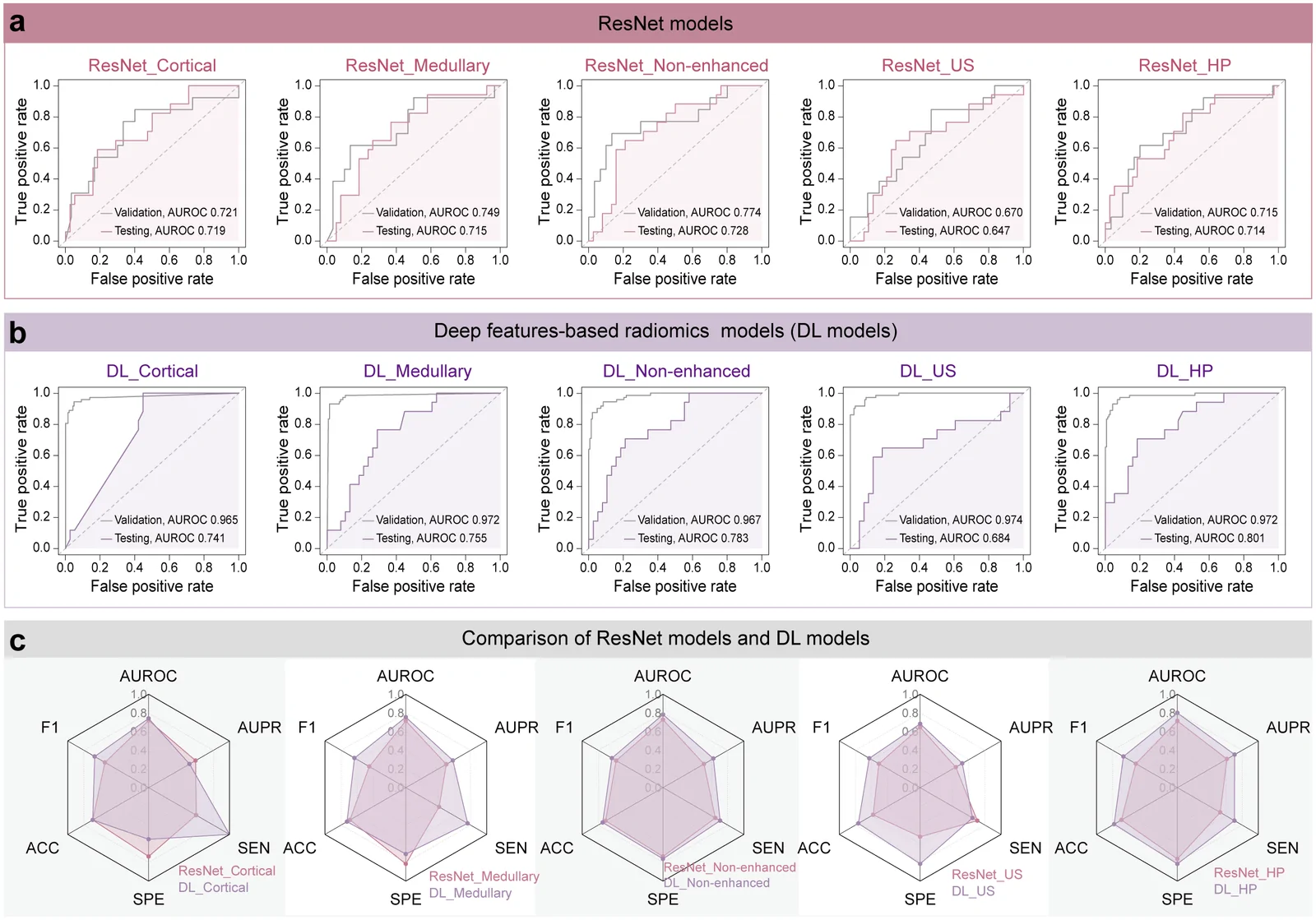
Classification performance of single-modality deep learning-based models in the validation and independent testing datasets. **(a)** Receiver operating characteristic (ROC) curves of the five end-to-end ResNet models on both the validation and independent testing datasets. **(b)** ROC curves of the five deep features-based radiomics (DL) models under identical validation and independent testing dataset splits. **(c)** Radar plots showing the comparison of the classification performance of the ResNet models and the DL models on the testing dataset.

Five deep features-based radiomics (DL) models were developed. While performance slightly decreased in the testing set compared to validation, the DL_HP model achieved the best performance (AUROC = 0.801). Notably, radar plots indicate that deep features-based radiomics generally outperformed the corresponding raw ResNet models across quantitative metrics (**Figure 4**; **Table 2**), highlighting the superiority of the combined DL-radiomics approach.

#### 3.3.4 Single-modality fusion models

**Figure 5:**
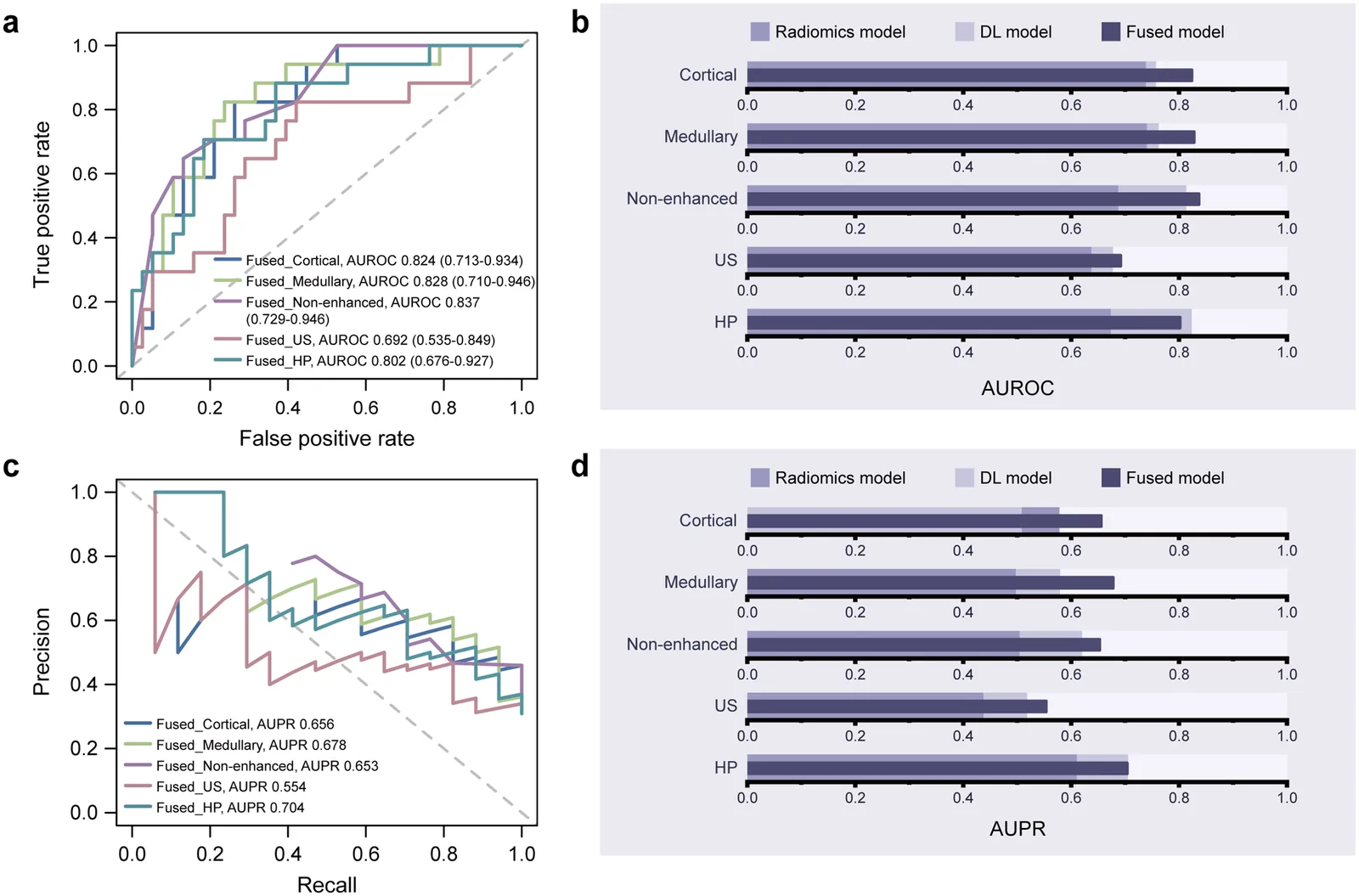
Classification performance of five single-modality fusion models in the independent testing dataset. **(a)** Receiver operating characteristic (ROC) curves of the five single-modality fusion models. **(b)** Comparison of AUROC for the radiomics model, deep features-based radiomics (DL) model, and fused model. **(c)** Precision-recall (PR) curves of the five single-modality fusion models. **(d)** Comparison of AUPR for the radiomics model, DL model, and fused model.

Single-modality fusion models were developed via stacked generalization. These models consistently improved classification performance over individual base models (**Figure 5**; **Table 3**). Specifically, the Fused_Non-enhanced model achieved the highest AUROC (0.837), surpassing both its corresponding radiomics and DL counterparts. This underscores the potential of integrating machine learning and deep learning perspectives to mine lesion information more robustly.

**Table 3:** Classification performance of fusion models on the independent testing dataset.

Model	AUROC (95% CI)	AUPR	ACC	F1	SEN	SPE	PRE
Single-modality fusion models							
• Fused_Cortical	0.824 (0.713 – 0.934)	0.656	0.691	0.622	0.824	0.632	0.500
• Fused_Medullary	0.828 (0.710 – 0.946)	0.678	0.782	0.700	0.824	0.763	0.609
• Fused_Non-enhanced	0.837 (0.729 – 0.946)	0.653	0.800	0.667	0.647	0.868	0.688
• Fused_US	0.692 (0.535 – 0.849)	0.554	0.655	0.596	0.824	0.579	0.467
• Fused_HP	0.802 (0.676 – 0.927)	0.704	0.764	0.629	0.647	0.816	0.611
Multi-modality fusion models							
• Multi-modality radiomics	0.858 (0.761 – 0.955)	0.744	0.764	0.683	0.824	0.737	0.583
• Multi-modality DL	0.924 (0.853 – 0.995)	0.873	0.873	0.811	0.882	0.868	0.750
• Multi-modality radiomics-DL	0.967 (0.929 – 1)	0.937	0.909	0.865	0.941	0.895	0.800
• Multi-modality fusion (i.e., fusion of single-modality fused models)	0.957 (0.910 – 1)	0.921	0.891	0.842	0.941	0.868	0.762

#### 3.3.5 Multi-modality fusion models

**Figure 6:**
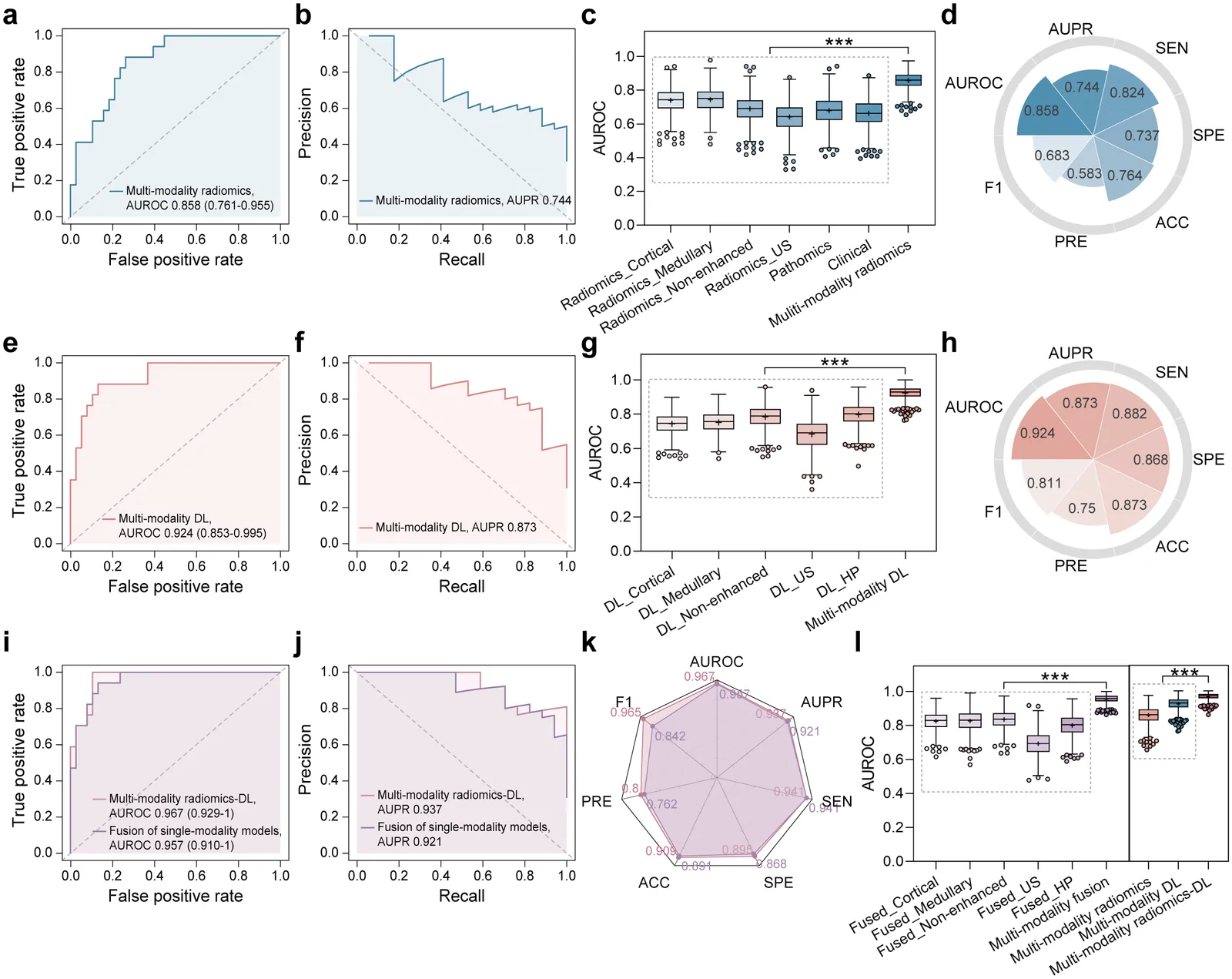
Multi-modality fusion models construction and performance. Performance of the multi-modality radiomics model **(a–d)**, multi-modality DL model **(e–h)**, and multi-modality radiomics-DL model **(i–l)** are visualized. ROC curves **(a, e, i)** and PR curves **(b, f, j)** of models in the independent testing dataset. **(c)** AUROC of the multi-modality radiomics model compared with that of six single-modality radiomics models. Quantitative metrics of the multi-modality radiomics **(d)**, multi-modality DL model **(h)**, multi-modality fusion model, and the multi-modality radiomics-DL model **(k)** in predicting the postoperative recurrence in the independent testing dataset are plotted. **(g)** AUROC of the multi-modality DL model compared with that of five single-modality DL models. **(l)** AUROC of the multi-modality fusion model compared with that of five single-modality fused models, and the multi-modality radiomics-DL model compared with multi-modality radiomics and multi-modality DL models. The AUROC values in c, g, l are shown in box-and-whisker plots, in which the line and the plus sign represent the median and the mean values, respectively. The whiskers range from 2.5th to 97.5th percentile, and points below and above the whiskers are drawn as individual dots. Statistical analyses were performed using Kruskal-Wallis H tests followed by Dunn’s multiple comparisons tests, with n = 1000 replicates per condition. The asterisks represent two-tailed adjusted P values, with ***P < 0.001.

To leverage complementary information, multi-modality fusion models were constructed (**Figure 6**; **Table 3**). The multi-modality radiomics model and multi-modality DL model achieved testing AUROCs of 0.858 and 0.924, respectively, significantly outperforming single-modality approaches. Modality ablation indicated that CT and HP were core drivers for radiomics, while CT, HP, and US were essential for DL performance (**Supplementary Table 6**). Ultimately, the integrated multi-modality radiomics-DL model attained the highest predictive performance with an AUROC of 0.967 (**Figure 6**), demonstrating the superiority of incorporating multi-modal information for accurate classification.

## 4 Discussion

AI models that incorporate multi-omics information have crucial values for ccRCC postoperative recurrence risk prediction in clinical practice. This retrospective multicenter study develops and validates a multimodal fusion prediction model by integrating patient demographic characteristics, laboratory tests indices, ccRCC radiological characteristics, ultrasonographic images, three-phase CT scans and histopathological sections. Results demonstrate that the multi-modality radiomics-DL model achieves the best predicting performance with an AUROC value of 0.967 (95% CI: [0.929-1]) in the independent testing dataset. Notably, this study validated the model using multicenter, multi-scanner data: Hospital A used five different CT scanners, Hospital B used three scanners, and Hospital C used a Siemens Definition Flash scanner not included in the training dataset. The consistent high performance of the model across these diverse devices confirms its robustness to scanner-related variability, which is critical for clinical translation.

Ficarra et al. (30) demonstrated that tumor necrosis, as shown by the Mayo Clinic Staging, Size, Grading and Necrosis (SSIGN) scoring system, is an important prognostic factor in the clinical management of patients with ccRCC. This finding is consistent with the results of the present study. Besides, the present study also suggests that tumor size plays a pivotal role in the risk of recurrence in patients with RCC, with larger tumors correlating with a higher probability of recurrence. Feng Z et al. (31), who developed a radiomics model demonstrated superior predictive performance with higher time-dependent AUCs (1-year: 0.882 vs. 0.781; 2-year: 0.865 vs. 0.762; 3-year: 0.793 vs. 0.797; all p < 0.05); What’s more, a study by Li et al. (32) also demonstrated that IPAT 5 mm radiomics model demonstrated superior predictive performance for tumor recurrence (C-index: 0.924 vs. 0.915-0.923 in the IVS; 0.952 vs. 0.920-0.944 in the EVS). Zine-Eddine et al. (33).employed CT images and clinical factors to predict the likelihood of distant metastases, the radiomics clinical model, which achieved an iAUC of 0.81 (95% CI: 0.76-0.85) in the training set and 0.78 (95% CI: 0.69-0.88) in the test set.

In this study, both the multi-modality radiomics-DL model and the multi-modality fusion model demonstrate superior prognostic performance compared to previous studies. The improved predictive performance can be attributed to the complementary nature of the information provided by different data modalities (34). For example, radiological scans and pathology specimens depict tumors spatially at varying scales, thus providing insights into distinct aspects of tumor biology (35). Critically, the observed performance inconsistency across radiomics, ResNet, and DL models is not spurious but reflects inherent differences in how each method captures multi-dimensional tumor heterogeneity: radiomics extracts handcrafted, interpretable features (e.g., texture entropy) that reflect stable macro-scale morphology, while ResNet captures high-level, non-linear spatial patterns (e.g., subtle micro-texture variations) that are difficult to quantify manually. DL models bridge these by balancing complex patterns with standardized quantification, confirming that no single method suffices for the biological complexity of ccRCC postoperative recurrence. The image features extracted by machine learning and deep learning may also be complementary, enabling enriched representation of tumor characteristics from different perspectives and at different levels. Integration of relevant information from multiple modalities finally enhanced the overall prediction performance and generalization ability of models. This complementarity validates the rationale for multi-method fusion, as evidenced by the superior AUROC (0.967) of our multi-modality radiomics-DL model, which mitigates individual limitations like radiomics’ feature sparsity or ResNet’s overfitting risk.

To further improve the predictive performance of models, an exploration into the process of data fusion is conducted. All fusion models employed a stacked ensemble strategy with explicit technical details: optimized base models generated “recurrence probability” for the entire primary dataset, which were used as meta-features. These meta-features were fed into a meta-classifier to output the final prediction, rather than simple averaging or concatenation of probabilities. This design ensures robust representation of predictions and outperforms conventional feature concatenation (36). Importantly, the independent testing dataset was isolated from all training-related steps, eliminating data leakage and ensuring the fusion strategy’s reproducibility. The fusion’s efficacy stems directly from harnessing the inconsistent but complementary outputs, which capture distinct tumor aspects (e.g., macro *vs.* micro scales), enhancing biological relevance.

Compared with recently published AI models for ccRCC recurrence prediction, our framework offers distinct advantages and clear incremental clinical value. Most existing studies have relied on single-modality data, such as CT-based radiomics alone (which captures macro-level tumor heterogeneity) or digital pathology-based deep learning alone (which focuses on micro-level cellular features). While these models perform well, they inevitably miss complementary prognostic dimensions. By contrast, our model synergizes three-phase CT, ultrasonography, whole-slide pathological images, and baseline clinical records. This comprehensive integration delivers substantial incremental value: ultrasonography provides real-time tissue stiffness and perfusion indicators, CT captures 3D spatial heterogeneity, and pathomics details the tumor microenvironment. Our results demonstrate that fusing these complementary streams yields a more robust and superior predictive performance than any single-modality counterpart or traditional clinical staging systems alone, providing clinicians with a more precise tool for personalized risk stratification.

The present study’s strength, in comparison with extensively studies, lies in its ability to efficiently integrate multimodal data. Recent studies have begun to explore the use of multimodal data to predict risk stratification and treatment response in a variety of cancers (37–39). However, To the best of our knowledge, there is no previous studies have utilized multimodal data for predicting the risk of postoperative recurrence in ccRCC patients. This integration provides practical clinical value: radiomics models offer a pragmatic choice for resource-limited settings, while DL or fusion models deliver higher accuracy for high-risk cases, optimizing personalized treatment. Although multimodal data is used in present study, additional data from cancer biomarkers, gene expression, and metabolomics can be incorporated in the future. The incorporation of these additional data sources has the potential to enhance the accuracy of models in predicting the postoperative recurrence risk in ccRCC patients, thereby optimizing personalized treatment strategies.

Regarding clinical translation, our multimodal framework can be seamlessly integrated into routine post-operative workflows without adding clinical or financial burdens. The required data—including three-phase CT, ultrasound, and histopathological WSIs—are already standard-of-care clinical inputs collected within 1 to 2 weeks after nephrectomy. Computationally, the fully trained model does not require high-performance clusters; it can run on a standard hospital workstation equipped with a single consumer-grade GPU. Once WSIs are digitalized (~5–10 minutes) and radiological images are retrieved from the Picture Archiving and Communication System (PACS), the inference and risk prediction process takes less than 5 minutes per patient. This extremely short turnaround time ensures that personalized risk stratification can be ready well before post-operative multidisciplinary team (MDT) meetings, facilitating timely and tailored follow-up or adjuvant therapy planning.

In terms of model interpretability, while our radiomics and pathomics features were selected and weighted using transparent, inherently explainable linear models (such as LASSO and logistic regression), the deep learning components for image feature extraction remain relatively “black-box.” To enhance clinicians’ trust and facilitate smooth clinical integration, future iterations of our platform will incorporate established explainability frameworks. Specifically, we plan to implement Gradient-weighted Class Activation Mapping (Grad-CAM) to visually highlight the exact histopathological sub-regions driving the ResNet predictions, as well as SHapley Additive exPlanations (SHAP) to quantitatively decompose the contribution of each clinical, radiological, and pathological feature to the final risk score.

Several limitations of this study are addressed here. First, patients who died without tumor recurrence within 5 years were excluded from the main analysis to focus on postoperative recurrence events and reduce potential interference from non-recurrence-related death. However, death without recurrence represents a competing event for tumor recurrence, and such exclusion may introduce selection bias. Second, although Hospital C served as an independent validation cohort, all participating centers belong to the same geographic region. This regional concentration might limit the generalizability of our model to cohorts with diverse demographics or different imaging/pathology protocols. Future validation using geographically diverse or international cohorts is warranted to establish broader clinical applicability. Third, although the model was trained and validated on patients from three independent centers, the independent external testing cohort remains relatively small (55 patients, 17 recurrence events). This limited cohort size may restrict the precision and stability of our multi-modality model’s performance estimates. Consequently, a larger, multi-institutional cohort is required in future studies to further validate our framework. Fourth, some clinical variables, such as drinking history, were defined simplistically as binary records and showed severe imbalance across the three centers. This imbalance suggests potential retrospective documentation bias and confounding, meaning the role of drinking in predicting recurrence must be interpreted with clinical caution. Fifth, although CT images employed nnU-Net to develop a segmentation model for automated segmentation, ultrasound images and pathology slice images still rely on experts to manually sketch ROIs in the images. Sixth, given its retrospective design, the present study may be subject to selection bias during patient recruitment. Last, bioinformatic analysis is not performed between two separating groups to explore the underlying biochemical mechanisms of the final predictive model.

The multi-modality radiomics-DL model is introduced as a novel and effective non-invasive approach for predicting the risk of 5-year postoperative recurrence in ccRCC patients. This advancement underscores the potential of such models to support clinical decision-making and promote personalized treatment strategies for ccRCC patients.

## Data Availability

The data analyzed in this study is subject to the following licenses/restrictions: The dataset contains private patient health information and is protected by ethical review requirements, patient privacy regulations, and institutional data management policies. Therefore, the raw data cannot be made publicly available. Access to the data is restricted and only permitted with approval from the ethics committee and relevant institutional authorities. Requests to access these datasets should be directed to Dataset access requests should be sent to XiangShui Meng at mengxiangshui2021@126.com, subject to ethical and institutional approval.
