# Supplementary material for: Multimodal deep learning for prediction of postoperative recurrence in clear cell renal cell carcinoma: a clinical-radiologic-pathologic approach
Source: Front Oncol. 2026 Aug 6;16:1831252. doi: 10.3389/fonc.2026.1831252 (PMC13489899; doi:10.3389/fonc.2026.1831252)
Supplement: Supplementary file 1 [file DataSheet1.doc]

**Supplementary Information**

**Participant Recruitment and Statistical Power**

**Inclusion and Exclusion Criteria**

Participants were considered eligible if they fulfilled the following criteria: (1) age ≥ 18 years with pathologically diagnosed ccRCC; (2) underwent radical or partial nephrectomy; (3) availability of high-resolution three-phase CT sequences (non-enhanced, cortical, medullary), US images, and H&E-stained WSI; and (4) comprehensive clinical information (demographics, laboratory measurements).

Exclusion criteria were: (1) history of other malignancies; (2) absence of CT/US/HP data; (3) poor image quality (e.g., motion artifacts, torn tissues); (4) loss to follow-up; and (5) death without recurrence within 5 years post-surgery.

**Sample Size and Power Analysis**

The primary dataset comprises 215 participants (143 recurrence-free, 72 recurrence). With a positive case rate of ~33%, the study achieves 93% power to detect an AUROC of 0.90 (vs. null hypothesis 0.65) at α = 0.05 (two-sided), ensuring statistical robustness for the multi-modality model evaluation.

**Data Acquisition Details**

**CT Scanning Specifications**

Hospital A utilized General Electric, Philips, Siemens, Canon, and Toshiba 512-row detectors. Hospital B used GE Revolution CT (256-slice), Siemens SOMATOM Definition, and SOMATOM Force. Hospital C used Siemens Definition Flash CT. Specific scanning parameters are detailed in Supplementary Table 1. CT Image quality was evaluated by two radiologists (8 and 10 years experience), with arbitration by a senior radiologist (20 years experience).

**Ultrasound Specifications**

US examinations utilized MyLab™ (Esaote, Italy) and Resona 7 (Mindray, China) devices. Quality was assessed by two sonographers (10 years experience each), arbitrated by a senior sonographer (18 years experience).

**Histopathological Scanning**

H&E slides were scanned using an SQS-120P scanner (Shenzhen Shengqiang Technology) at 20×. A pathologist (9 years experience) verified slide quality, excluding cases with artifacts (tears, folds, discoloration).

**Preprocessing Parameters**

CT: Resampling to 1×1×1 mm³; gray-level normalization (±3 sigma); discretization (64 bins); z-score standardization.

US: Gaussian filtering; histogram equalization.

HP: Macenko normalization (α=0.15, β=0.01) to standardize H&E staining; Gaussian filtering; conversion to grayscale.

**Automated CT Segmentation (nnU-Net)**

A secondary nnU-Net model was trained on cortical phase images (80% training, 20% testing). Performance metrics included Dice similarity coefficient (DSC), Jaccard index, Hausdorff distance (HD), and HD95. This model served as a feasibility test for automation but was not used for the primary prognostic analysis.

**Annotation Software and Standards**

Registration: Rigid registration aligned multi-phase CTs; accuracy verified by DSC > 0.80.

US: Manual delineation using ITK-SNAP on the largest cross-section.

HP: Tumor cores annotated in ImageScope; patches sampled strictly from viable tumor regions.

**Model Construction Details**

**1. Radiomics Feature Extraction and Definition**

Radiomics feature extraction was performed using the uAI research portal (uRP, United Imaging Intelligence) calling PyRadiomics (version 2.11) with Python (version 3.7).

CT Features (n=2264 per phase): Extracted from 3D ROIs. Included first-order intensity statistics, 3D shape features, and texture features. Multiple filters were applied to capture global tumor heterogeneity.

US Features (n=1899): Extracted from 2D ROIs of the largest cross-section. Included first-order statistics, 2D shape features, and texture features, using the same filter set as CT.

Pathomics (HP) Features (n=93): Extracted from 256×256 pixel patches in the tumor core. Included first-order statistics and texture features reflecting cellular-level morphological and staining heterogeneity (no additional filters applied).

Definitions: Mathematical definitions adhere to the Image Biomarker Standardization Initiative (IBSI) guidelines and are available at the PyRadiomics documentation (https://pyradiomics.readthedocs.io/en/latest/radiomics.html).

**2. Feature Selection Strategy**

Feature selection was conducted on the primary dataset via 5-fold cross-validation (CV).

Stability Check: Intra-observer agreement was assessed using the intraclass correlation coefficient (ICC); only features with ICC > 0.75 were retained.

Dimensionality Reduction: Least Absolute Shrinkage and Selection Operator (LASSO) regression or F-test techniques were employed.

Constraints: Adhering to Harrell's guideline (10 events per predictor parameter), the final model dimension was strictly limited to a maximum of six features per model.

Robustness: Features recurring above a specific threshold across 5-fold experiments were selected.

**3. Machine Learning Classifiers and Preprocessing**

Normalization: Selected features were standardized using various methods including z-score scaler, min-max scaler, max-abs scaler, quantile transformer, and L1 normalization.

Classifiers: The following algorithms were evaluated to identify the best-performing model for each modality: Logistic Regression (LR), Random Forest (RF), Bagging Decision Tree (DT), and Gradient Boosting Decision Tree (GBDT).

**4. Deep Learning Architecture (ResNet34)**

Network Structure: The ResNet34 architecture comprises 34 convolutional layers. It begins with a convolutional layer and max pooling, followed by four building layers containing 3, 4, 6, and 3 residual modules respectively. A global average pooling layer precedes the fully connected layers and Softmax output.

Input Processing: All images (CT/US/HP) were resized to 256×256 pixels. Pixel values were normalized to [0,1] (divided by 255). The normalized image and its binary ROI mask were concatenated as dual-channel inputs.

Training Hyperparameters:

Weights: Pre-trained on ImageNet (transfer learning).

Loss Function: Cross-entropy loss.

Optimizer: Adam.

Batch Size: 16.

Learning Rate: 0.0001.

Strategy: Early stopping was implemented on the validation set (20% of primary data) to prevent overfitting.

**5. Deep Features-based Radiomics (DL) Workflow**

Deep features were extracted from the penultimate fully connected layer of the trained ResNet34 models.

Selection Process: Deep features were extracted from the 80% training subset for LASSO selection.

Validation: Features for the validation and independent testing sets were extracted using the fixed ResNet model but were not involved in the selection process to prevent data leakage.

**6. Fusion Model Design (Stacked Ensemble)**

Meta-features: The "probability of postoperative recurrence = 1" output from base models.

Base Model Optimization:

Radiomics Base: Trained on the entire primary dataset after 5-fold CV optimization.

ResNet/DL Base: Trained on the 80% training subset after hyperparameter tuning on the 20% validation subset.

Meta-classifier: A logistic regression model trained on the meta-features derived from the primary dataset.

**Model Naming Conventions**

To ensure clarity, models are labeled based on their analytical pipeline and data modality:

Radiomics_[Modality]: Models using the standard radiomics pipeline (handcrafted feature extraction + ML). Includes "Pathomics" and "Clinical" models.

ResNet_[Modality]: End-to-end deep learning models based on ResNet34.

DL_[Modality]: Deep features-based radiomics models (Deep features extracted from ResNet + ML).

Fused_[Modality]: Single-modality fusion models (e.g., Fused_Cortical = Radiomics_Cortical + DL_Cortical).

Multi-modality Models: Named according to the source of integrated probabilities (e.g., Multi-modality Radiomics-DL model).

**Statistical Analysis Details**

Descriptive Statistics: Normally distributed variables are presented as mean ± standard deviation; non-normal variables as median (25th, 75th percentiles). Categorical variables are expressed as counts and percentages.

Feature Correlation: Correlations of selected features in single-modality radiomics models were assessed via Pearson or Spearman methods.

Specific Model Comparisons: The Kruskal-Wallis H test with Dunnett’s post-hoc test was specifically applied to compare:

Multi-modality radiomics model vs. six single-modality radiomics models.

Multi-modality DL model vs. five single-modality DL models.

Multi-modality fusion model vs. single-modality fusion models.

Multi-modality radiomics-DL model vs. constituent multi-modality models.

Software & Plotting: R packages included ROCR and fbroc. Figures were generated using GraphPad Prism 9, OriginPro 2021, and Adobe Illustrator 2023.

**Supplementary Results**

**1. Detailed Analysis of Patient Population and Risk Factors**

As referenced in the main text, significant heterogeneity was observed across the three medical centers. Statistical analysis revealed significant differences in 14 variables: smoking (P = 0.017), drinking (P < 0.001), blood glucose (P < 0.001), white blood cells (P = 0.006), neutrophils (P = 0.002), basophils (P < 0.001), packed cell volume (P = 0.025), platelets (P = 0.016), epithelial cells (P = 0.004), hemorrhage (P < 0.001), necrosis (P < 0.001), lobulation (P = 0.001), lymphadenopathy (P = 0.001), and capsule involvement (P < 0.001).

Regarding the univariate logistic regression analysis for recurrence risk factors, detailed statistical outcomes for the five significant variables are as follows: maximum diameter of ccRCC (P < 0.001, OR 2.12, 95% CI 1.60-2.80), arteriovenous thrombosis (P < 0.001, OR 1.61, 95% CI 1.25-2.07), lymphadenopathy (P = 0.012, OR 1.36, 95% CI 1.07-1.73), necrosis (P = 0.035, OR 1.30, 95% CI 1.02-1.67), and drinking (P = 0.042, OR 1.29, 95% CI 1.01-1.66).

**2. Evaluation Metrics for Automatic Segmentation**

Beyond the Dice Similarity Coefficient (DSC) reported in the main text, the segmentation model was evaluated using error-related metrics. The model demonstrated low values for Relative Absolute Volume Difference (RAVD), Hausdorff Distance (HD), 95% Hausdorff Distance (HD95), and Average Surface Distance (ASD), further confirming the reliability of the nnU-Net architecture for this task (refer to Supplementary Table 3).

**3. Feature Selection for Radiomics Models**

Based on the five-fold cross-validation and thresholding method, the specific dimensions of selected features for the six single-modality radiomics models were: 5 features for Non-enhanced, 6 for Cortical, 6 for Medullary, 6 for US, 5 for HP, and 4 for Clinical models. Correlation analyses for these selected features are visualized in Supplementary Figure 4, where significant correlations are marked.

**4. Detailed Performance of Deep Learning and Fusion Models**

Single-modality ResNet Models: In the independent testing dataset, the 95% confidence intervals (CI) for the ResNet models were as follows: ResNet_Non-enhanced (AUROC 0.728, 95% CI 0.587-0.868) and ResNet_US (AUROC 0.647, 95% CI 0.486-0.808).

Deep Features-based Radiomics Models: In the validation dataset, these models showed robust performance with AUROC values exceeding 0.960. In the testing dataset, the DL_HP model achieved an AUROC of 0.801 (95% CI 0.678-0.924), while the DL_US model achieved 0.684 (95% CI 0.512-0.856).

Single-modality Fusion Models: The meta-classifier for these models was trained on the primary dataset (n = 215) with rigorous separation from the testing set. Detailed performance metrics in the testing set included: Fused_Non-enhanced (AUROC 0.837, 95% CI 0.729-0.946) and Fused_US (AUROC 0.692, 95% CI 0.535-0.849). AUPR visualization plots (Figures 5c ~ 5d) confirmed that four out of five fusion models (excluding Fused_HP) outperformed their individual counterparts.

**5. Modality Ablation Analysis Details**

Multi-modality Radiomics: Ablation studies indicated that the inclusion of US or clinical variables reduced performance due to data redundancy. The core performance drivers were identified as CT and HP modalities.

Multi-modality DL: The synergistic value of combining CT, HP, and US was confirmed quantitatively. The tri-modality combination (AUROC 0.924) outperformed subsets such as CT alone (AUROC 0.842) or the dual-modality CT + HP combination (AUROC 0.898), establishing all three as essential for optimal Deep Learning performance.

The final integrated Multi-modality Radiomics-DL model achieved an AUROC of 0.967 (95% CI 0.929-1), representing the peak performance across all constructed models.

**1. Supplementary Figures**

**Supplementary Figure 1. Statistical power calculated by G*Power software.** The power reached 0.931 for the given parameters (α, sample sizes, means, and σ).

**Supplementary Figure 2. Segmentation performance of the automatic nnU-Net model.** Six quantitative metrics are visualized, including **(a)** Dice similarity coefficient (DSC), **(b)** Jaccard index (JC), **(c)** relative absolute volume difference (RAVD), **(d)** Hausdorff distance (HD), **(e)** 95% HD (HD95), and **(f)** average surface distance (ASD). The numbers in the plots are the mean values.

**Supplementary Figure 3. The number of times for each feature in the five-fold cross-validation, selected for constructing radiomics models.** Given the feature selection method and parameters, each fold selects a different set of features based on the corresponding training dataset, and ultimately, we select features that occur more than a certain threshold.

**Supplementary Figure 4. Correlation analyses of selected features in six single-modality radiomics models.**

**Supplementary Figure 5. Confusion matrices of four multi-modality fusion models in the independent testing dataset.**

**2. Supplementary Tables**

All data were collected from three centers, as Jinan Branch of Hospital A, Hospital B, and Hospital C.

**Supplementary Table 1. Scanning parameters for renal CT examination.**

| Parameter | Hospital A | Hospital B | Hospital C |
| --- | --- | --- | --- |
| Contrast agent | Ultravist 300, Omnipaque 300 | Ultravist 300, Iopamidol | Omnipaque 300 |
| Injection rate of contrast agent | 2.8 mL/s, 3 mL/s | 2.8 mL/s, 3 mL/s | 3 mL/s |
| Tube current | Automatic modulation | Automatic modulation | Automatic modulation |
| Tube voltage | 100~120 kV | 100~120 kV | 100~120 kV |
| Slice thickness | 5 mm, 1 mm | 5 mm, 1 mm | 1 mm |
| Matrix | 512 × 512 | 512 × 512 | 512 × 512 |
| Convolution kernel | Br40, 130f | Br40, 130f | Br40 |

**Supplementary Table 2. Univariate logistic regression used to identify independent risk factors for metastasis.** The right panel shows the OR (95% CI) distribution of the 11 variables.

**Supplementary Table 3. Segmentation performance of the automatic nnU-Net model. Continuous variables that do not adhere to a normal distribution are displayed as the median (25^th^, 75^th^ percentiles).**

| Variables | Training | Testing |
| --- | --- | --- |
| DSC | 0.931 (0.903, 0.948) | 0.910 (0.867, 0.933) |
| JC | 0.872 (0.823, 0.901) | 0.834 (0.765, 0.875) |
| RAVD | 0.000 (-0.048, 0.035) | -0.002 (-0.075, 0.104) |
| HD | 5.385 (3.606, 8.335) | 8.602 (4.472, 12.369) |
| HD95 | 1.000 (1.000, 2.000) | 2.000 (1.000, 3.742) |
| ASD | 0.300 (0.226, 0.417) | 0.482 (0.317, 0.846) |

**Supplementary Table 4. Parameters for constructing six single-modality radiomics models.**

| Model | No. of input features | No. of features retained after ICC | Feature selection method | No. of selected features | Data preprocessor | Classifier | Hyperparameters in modeling |
| --- | --- | --- | --- | --- | --- | --- | --- |
| Radiomics_  Cortical | 2264 | 1685 (74.4%) | LASSO, α = 0.08, ≥ 3 times | 5 | Quantile transformer | Bagging DT | - Max depth: 4 - Max features: 1 - Max samples: 1 - Number of base estimators: 100 |
| Radiomics_  Medullary | 2264 | 1592 (70.3%) | LASSO, α = 0.07, ≥ 3 times | 6 | Max-abs scaler | Bagging DT | - Max depth: 4 - Max features: 1 - Max samples: 1 - Number of base estimators: 100 |
| Radiomics_  Non-enhanced | 2264 | 1611 (71.2%) | LASSO, α = 0.04, 5 times | 6 | Z-score scaler | LR | - Regularization parameter C: 1.0 - Class weight: None - Penalty parameter: l2 - Tolerance: 0.0001 |
| Radiomics_  US | 1899 | 1328 (69.9%) | LASSO, α = 0.04, ≥ 4 times | 6 | Min-max scaler | LR | - Regularization parameter C: 1.0 - Class weight: None - Penalty parameter: l2 - Tolerance: 0.0001 |
| Pathomics | 93 | 75 (80.6%) | LASSO, α = 0.03, ≥ 4 times | 5 | L1 normalization | RF | - Class weight: None - Criterion: Gini - Max depth: 2 - Min samples per leaf: 1 - Min samples per split: 2 - Number of base estimators: 100 |
| Clinical | 41 | Not applicable | F test, *P* < 0.05, 5 times | 4 | Z-score scaler | Gradient boosting DT | - Criterion: friedman_mse - Learning rate: 0.1 - Max depth: 3 - Min samples per leaf: 1 - Min samples per split: 2 - Number of base estimators: 100 |

**Supplementary Table 5. Classification performance of the single-modality models on the validation dataset.**

| Model | AUROC (95% CI) | ACC | F1 | SEN | SPE | PRE |
| --- | --- | --- | --- | --- | --- | --- |
| Radiomics models |  |  |  |  |  |  |
| - Radiomics_Cortical | 0.796 (0.676 – 0.939) | 0.763 | 0.681 | 0.768 | 0.763 | 0.623 |
| - Radiomics_Medullary | 0.759 (0.620 – 0.918) | 0.758 | 0.687 | 0.807 | 0.733 | 0.606 |
| - Radiomics_Non-enhanced | 0.790 (0.664 – 0.937) | 0.730 | 0.663 | 0.793 | 0.700 | 0.571 |
| - Radiomics_US | 0.726 (0.582 – 0.892) | 0.730 | 0.618 | 0.682 | 0.755 | 0.629 |
| - Pathomics | 0.642 (0.471 – 0.833) | 0.642 | 0.526 | 0.598 | 0.666 | 0.489 |
| - Clinical | 0.744 (0.603 – 0.906) | 0.712 | 0.611 | 0.669 | 0.736 | 0.589 |
| ResNet models |  |  |  |  |  |  |
| - ResNet_Cortical | 0.721 (0.543 – 0.899) | 0.721 | 0.538 | 0.538 | 0.800 | 0.538 |
| - ResNet_Medullary | 0.749 (0.577 – 0.920) | 0.721 | 0.571 | 0.615 | 0.767 | 0.533 |
| - ResNet_Non-enhanced | 0.774 (0.607 – 0.942) | 0.791 | 0.640 | 0.615 | 0.867 | 0.667 |
| - ResNet_US | 0.670 (0.496 – 0.844) | 0.628 | 0.385 | 0.385 | 0.733 | 0.385 |
| - ResNet_HP | 0.715 (0.543 – 0.888) | 0.721 | 0.500 | 0.462 | 0.833 | 0.545 |
| Deep features-based radiomics models (DL models) | | | | | | |
| - DL_Cortical | 0.965 (0.940 – 1) | 0.944 | 0.919 | 0.917 | 0.958 | 0.924 |
| - DL_Medullary | 0.972 (0.958 – 1) | 0.958 | 0.936 | 0.903 | 0.986 | 0.972 |
| - DL_Non-enhanced | 0.967 (0.952 – 1) | 0.944 | 0.913 | 0.876 | 0.979 | 0.956 |
| - DL_US | 0.974 (0.967 – 1) | 0.935 | 0.905 | 0.916 | 0.944 | 0.899 |
| - DL_HP | 0.972 (0.963 – 1) | 0.944 | 0.915 | 0.890 | 0.972 | 0.945 |

**Supplementary Table 6. Selection of the best multi-modality radiomics model and multi-modality DL model.** All metrics are calculated in the independent testing dataset.

| Model | AUROC (95% CI) | ACC | F1 | SEN | SPE | PRE |
| --- | --- | --- | --- | --- | --- | --- |
| Multi-modality radiomics models |  |  |  |  |  |  |
| - CT | 0.830 (0.720 – 0.939) | 0.764 | 0.683 | 0.824 | 0.737 | 0.583 |
| - CT + HP (best) | **0.858 (0.761 – 0.955)** | 0.764 | 0.683 | 0.824 | 0.737 | 0.583 |
| - CT + HP + US | 0.825 (0.708 – 0.942) | 0.727 | 0.667 | 0.882 | 0.658 | 0.536 |
| - CT + HP + US + Clinical | 0.816 (0.693 – 0.938) | 0.782 | 0.667 | 0.706 | 0.816 | 0.632 |
| Multi-modality DL models |  |  |  |  |  |  |
| - CT | 0.842 (0.733 – 0.951) | 0.764 | 0.683 | 0.824 | 0.737 | 0.583 |
| - CT + HP | 0.898 (0.815 – 0.980) | 0.818 | 0.722 | 0.765 | 0.842 | 0.684 |
| - CT + HP + US (best) | **0.924 (0.853 – 0.995)** | 0.873 | 0.811 | 0.882 | 0.868 | 0.750 |
